# Progress in Orthotopic Pig Heart Transplantation in Nonhuman Primates

**DOI:** 10.3389/ti.2024.13607

**Published:** 2024-09-27

**Authors:** Matthias Längin, Martin Bender, Michael Schmoeckel, Bruno Reichart

**Affiliations:** ^1^ Department of Anesthesiology, LMU University Hospital, LMU Munich, Munich, Germany; ^2^ Department of Cardiac Surgery, LMU University Hospital, LMU Munich, Munich, Germany; ^3^ Transregional Collaborative Research Center 127, Walter Brendel Centre of Experimental Medicine, LMU Munich, Munich, Germany

**Keywords:** orthotopic heart transplantation, xenotransplantation, organ perfusion, costimulation blockade, genetically-modified pig

## Abstract

Xenotransplantation of porcine hearts has become a promising alternative to human allotransplantation, where organ demand still greatly surpasses organ availability. Before entering the clinic, however, feasibility of cardiac xenotransplantation needs to be proven, ideally in the life supporting orthotopic pig-to-nonhuman primate xenotransplantation model. In this review, we shortly outline the last three decades of research and then discuss in detail its most recent advances. These include the genetic modifications of donor pigs to overcome hyperacute rejection and coagulation dysregulation, new organ preservation methods to prevent perioperative xenograft dysfunction, experimental immunosuppressive and immunomodulatory therapies to inhibit the adaptive immune system and systemic inflammation in the recipient, growth control concepts to avoid detrimental overgrowth of the porcine hearts in nonhuman primates, and lastly, the avoidance of porcine cytomegalovirus infections in donor pigs. With these strategies, consistent survival of 6–9 months was achieved in the orthotopic xenotransplantation model, thereby fulfilling the prerequisites for the initiation of a clinical trial.

## Introduction

Since the first orthotopic transplantation of a porcine heart into a baboon 30 years ago [1], there has been tremendous progress towards the ultimate goal - moving cardiac xenotransplantation (xHTx) into the clinic. In the beginning, most research focused on the non-life supporting heterotopic abdominal xHTx model [2–11], primarily to study immunosuppressive regimes to overcome hyperacute and acute rejection (reviewed in detail [12–14]). While xenograft survival in this model had steadily increased from several weeks to months and years, results in the life supporting orthotopic xHTx model were not encouraging: until 2017, most experiments lasted only a few days and many recipient animals were lost during the first 48 h [1, 2, 15–21]; maximum survival was 57 days [21]. These results were far from the recommendations devised by an expert committee of the International Society for Heart and Lung Transplantation (ISHLT) in 2000 [22]: as a prerequisite for a clinical application of xHTx, consistent 90-day survival of 60% of the animals in a life supporting xHTx model was deemed necessary. In the following 5 years, research in the field yielded many new insights, leading not only to improvements in both survival consistency and survival time [23–27], but also to the first clinical xenotransplantation of a porcine heart into a human [28].

In the following review, we specifically aim at a detailed overview on this recent period and highlight key findings that facilitated the progress of xHTx.

## First Decade (1994–2003)

**FIGURE 1:**
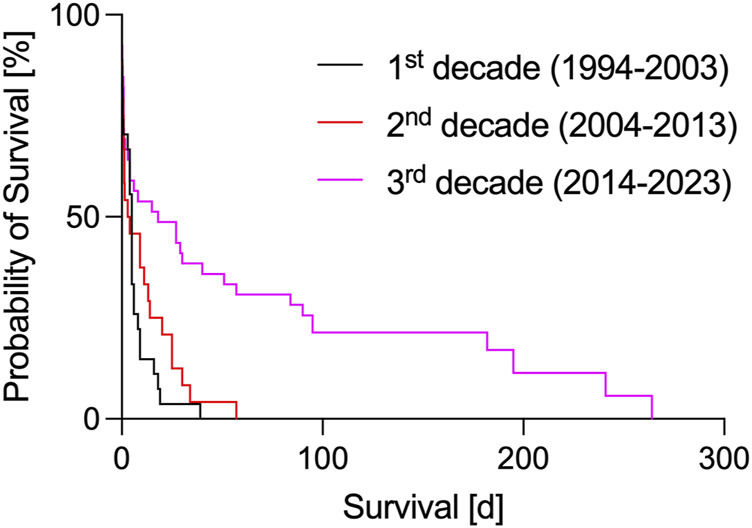
Survival after orthotopic pig-to-baboon cardiac xenotransplantations from 1994 to 2023. Data taken from [1, 2, 15–21, 23–27].

In the first decade of orthotopic xHTx, research in Loma Linda (CA, USA) and Cambridge (UK) focused on overcoming hyperacute and acute rejections. Survival rates where however short and inconsistent (Table 1; Figure 1) [1, 2, 15–17], with an overall median survival of 6.6 ± 1.6 (SEM) days and a maximum survival of 39 days [17]. At that time, the main cause for hyperacute rejection after porcine xHTX into non-human primates – the binding of preformed antibodies to the carbohydrate antigen galactose-α1,3-galactose (αGAL) on porcine endothelial cells – could not be avoided since cloning techniques to knock-out αGAL were not established in the pig [29]. By adding the human transgene *hDAF* (or *hCD55*) to the donor pig genome [30], complement activation following binding of anti-pig-antibodies to αGal epitopes could be attenuated, and graft survival was prolonged [2, 16, 17]. However, without further genetic modifications and the “standard” clinical immunosuppression of that time (i.a. cyclosporin A, methotrexate, cyclophosphamide, corticosteroids), expectations of a fast translation into the clinic could not be met.

**TABLE 1:** Orthotopic pig-to-baboon cardiac xenotransplantation experiments in the last three decades (1994–2023).

Year	Author	Donor genetics	Organ preservation	Immunosuppression	Immunomodulation	Growth inhibition	Survival
1994	Fukushima Nz [1]	WT	static	CsA, DSG	Immunadsorption		3 days, 4 days, 4 days, 5 days, 6 days, 6 days, 8 days, and 16 days
1998	Xu H [15]	WT	static	CsA, MTX, Cs, ATG	TLI		3 h, 18 days, 19 days
1998	Schmoeckel M [16]	hCD55	static	CsA, CyP, Cs			6 h, 6 h, 9 h, 10 h, and 18 h, 4 days, 5 days, 5 days, 5 days, and 9 days
1998	Waterworth PD [2]	hCD55	static	CsA, CyP, Cs			0 h, 0 h, 5 days, 5 days, 9 days
2000	Vial CM [17]	hCD55	static	CsA, CyP, Cs, MMF			39 days
2005	Brandl U [18]	hCD55	static	CyP, Cs, Tac, mTOR-I, ATG	aGAL polymer^a^		1 day, 30 h, 9 days, 25 days
2005	Brenner P [19]	hCD55	static	CsA, CyP, Cs, MMF			1 h, 11.1 days, 13.1 days, 20 days
2007	Brandl U [20]	hCD55^a^, hCD46^a^	static	CyP, Cs, Tac, mTOR-I, ATG, Rix^a^	aGAL polymer^a^		1 day, 9 days; 0 h, 30 h; 0 h, 20 h, 14 days, 25 days; 5.5 h, 9.5 h, 34 h, 3 days, 4 days
2008	McGregor CGA [21]	hCD46	static	Tac, mTor-I, ATG	aGAL polymer		34 days, 40 days, 57 days
2018	Längin M [23]	GGTA1-KO, hCD46, hTBM	static^a^, perfusion^a^	Cs, MMF, anti-CD40 ab^a^/anti-CD40L PAS Fab^a^, ATG, Rix	C1-I, anti-IL1R, anti-IL6R, anti-TNFa	mTOR-I^a^, ACE-I^a^, BB^a^	1 day, 1 day, 1 day, 3 days, 30 days; 4 days, 18 days, 27 days, 40 days; 51 days, 90 days, 90 days, 182 days, 195 days
2020	DiChiacchio L [24]	GGTA1-KO, CMAH-KO^a^, B4GALNT2-KO^a^, hCD55^a^, hCD46, hTBM^a^, hEPCR^a^, hCD47^a^, hHMOX1^a^, hvWF^a^	static	Cs, MMF, anti-CD40 ab, ATG, Rix	CVF		2 h, 7 h, 18 h, 22 h, 26 h, 40 h
2020	Reichart B [25]	GGTA1-KO, hCD46, hTBM	perfusion	Cs, MMF, anti-CD40 ab, ATG, Rix	C1-I, anti-IL1R, anti-IL6R, anti-TNFa	mTOR-I, ACE-I, BB	15 days, 27 days, 90 days, 90 days
2022	Mohiuddin MM [26]	GGTA1-KO, CMAH-KO^a^, B4GALNT2-KO^a^,hCD55^a^, hCD46^a^, hTBM^a^, hEPCR^a^,hCD47^a^,hHMOX1^a^, hvWF^a^	static^a^ (blood cardio), perfusion^a^	Cs, MMF, anti-CD40 ab, ATG, Rix	CVF^a^, C1-I^a^, anti-IL6R, anti-TNFa	GHR-KO^a^	6 h, 4 days, 29 days, 57 days; 12 h, 6 days, 8 days; 84 days, 95 days; 182 days, 264 days
2022	Cleveland DC [27]	GGTA1-KO, hCD55^a^, hCD46^a^, hTBM^a^	static (del Nido^a^)	Cs, mTOR-I, anti-CD40 ab, ATG, Rix	C1-I, anti-TNFa	BB^a^	3 h; 4 h, 90 days, 241 days

WT, wildtype; CsA, cyclosporine A; DSG, desoxyspergualin; MTX, methotrexate; Cs, corticosteroids; ATG, anti-thymocyte globulin; CyP, cyclophosphamide; MMF, mycophenolate mofetil; Tac, tacrolimus; mTOR-I, mTOR, inhibitor (sirolimus, temsirolimus); Rix, rituximab; anti-CD40 ab, anti-CD40 monoclonal antibody; anti-CD40L PAS Fab, PASylated anti-CD40L antibody fragment; TLI, total lymphoid irradiation; C1-I, C1-esterase inhibitor; anti-IL1R, interleukin 1 receptor blocker; anti-IL6R, interleukin 6 receptor blocker, anti-TNFα, tumor necrosis factor alpha inhibitor; ACE-I, angiotensin-converting enzyme inhibitor; BB, beta blockers; GHR-KO, growth hormone receptor knockout.

^a^ drug/genetic modification used only in some experiments of the respective study.

## Second Decade (2004–2013)

With the discovery of the αGAL epitope (reviewed in [31]), research groups in Rochester (MN, United States) and Munich (Germany) began infusing synthetic GAL oligosaccharides in recipients of orthotopic xHTx, with the aim of binding and inactivating anti-αGAL-antibodies, and finally preventing hyperacute rejection reactions [18, 20, 21]. As another strategy, immunoadsorption was explored [32–34]. The human complement-regulating transgene *hCD46* was introduced to counteract complement activation independent of antibody binding [35] and tested in orthotopic xHTx experiments [18, 21]. New clinically available immunosuppressants, such as mycophenolate mofetil, tacrolimus or rapamycin, were used, with and without B cell and T cell depletion (anti-thymocyte globulin (ATG), rituximab) for induction [18–21]. With these improvements, graft rejection could be delayed, and overall mean survival increased to 10.9 ± 2.9 (SEM) days (Table 1; Figure 1), with the longest single survivor reaching 57 days [21].

Although these results were promising, they still by far did not meet the prerequisites for a clinical study as defined by the ISHLT [22]. Especially puzzling was the fact, that the results after heterotopic abdominal pig-to-baboon xHTx experiments, with survivals of up to 179 days [5], were superior to those after orthotopic xHTx. The main culprit was “Perioperative Cardiac Xenograft Dysfunction” [36] (PCXD), a systolic organ failure that led to graft loss in up to 60% within the first 48 h after orthotopic transplantations, without any signs of rejection [35]. Possible explanations for PCXD included various xeno-specific factors, such as the use of young donor organs, inflammatory effects of preformed non-GAL antibodies, incompatibility between porcine and primate plasma; donor-specific factors, notably the sensitivity of the porcine heart to cardiopulmonary bypass and ischemia/reperfusion injury, were also discussed [reviewed in [36]].

As successful translation to the orthotopic pig-to-baboon model seemed more and more impossible, researchers focused on refining the heterotopic abdominal model; the question was raised, if further orthotopic xHTx experiments were at all needed for clinical application [37].

## Third Decade (2014–2023)

With the creation of knockout pigs lacking the gene coding for αGAL (*GGTA1*-KO) [38] and the addition of human thrombomodulin (*hTBM*) to overcome thrombotic microangiopathy due to interspecies coagulation incompatibilities [39], survival rates after heterotopic abdominal xHTx increased even further: In 2016, M. Mohiuddin reached a median graft survival of 298 days in five baboons (maximum survival 945 days) with triple genetically modified donor piglets (*GGTA1-KO*, *hCD46*, and *hTBM*) and an immunosuppression based on an experimental chimeric CD40/CD40L costimulation blockade (mouse/rhesus monkey clone 2C10R4) [11]. In two of these long-term experiments, graft rejection was intentionally triggered by discontinuation of the costimulation blockade, demonstrating the importance of this specific therapy.

By translating the knowledge gained from these heterotopic abdominal xHTx experiments and new approaches in organ preservation and growth inhibition, the field of orthotopic pig-to-baboon xHTx was stimulated by the work of the Munich group [23] and, subsequently, further advanced by the group of M. Mohiuddin in Baltimore (MD, United States) [26]. Overall mean survival in this decade increased to 51.8 ± 11.5 (SEM) days (Table 1; Figure 1), with the longest single survivor reaching 264 days [26]. And even more so, in 2020 the recommendations of the ISHLT [22] were finally fulfilled for the first time [25], paving the way for a first clinical trial. The most important advances of this last decade are now reviewed in more detail in the following.

## Genetic Modifications

The longest surviving grafts in recent orthotopic pig-to-baboon xHTx studies included at least the following three genetic modifications [23, 26, 27]: *GGTA1-KO* for the removal of the αGAL epitope, insertion of the complement regulator *hCD46*, and *hTBM* to overcome interspecies coagulation incompatibilities. With these minimum set of modifications, maximum survival times were 195 [23], 241 [27], and 264 days [26]. In the latter experiment, however, the porcine donor also carried several additional modifications: KOs of β-1,4-N-acetyl-galactosaminyl transferase 2 (*B4GALNT2-KO*), and growth hormone receptor (*GHR-KO*); additional transgenes included a second complement regulator protein (*hCD55*), the human endothelial protein C receptor (*hEPCR*) and the human signal regulatory protein alpha *hCD47*. It remains unclear to what extent these additional modifications contributed to graft survival.

For human recipients, KOs of the genes *B4GALNT2* and cytidine monophosphate-N-acetylneuraminic acid hydroxylase (*CMAH)* in addition to *GGTA1-KO* are important for xHTx because of preformed antibodies against their respective gene products N-glycolylneuraminic acid (Neu5Gc) and a glycan corresponding to the human Sd(a) blood group antigen (β4GAL) (“triple KO-pig,” reviewed in [40]). In the nonhuman primate (NHP) model, by contrast, *CMAH*-KO has been reported to be disadvantageous due to the presumed exposure of another, yet unknown xenoantigen [41, 42]. This is supported by results from Mohiuddin’s study, in which maximum survival of grafts with *CMAH-*KO was only 8 days [26].

In the last few years, a plethora of genetical modifications have been described, including deletion of genes coding for carbohydrate antigens and expression of complement and coagulation regulatory, as well as immunomodulatory proteins (reviewed in detail elsewhere [40]). Further studies are needed to better define which combination of additional modifications might prove beneficial for graft survival. Based on existing data [23, 26, 27], at least a minimum of one complement regulator and one coagulation regulatory protein in addition to *GGTA1-KO* seems to be required.

## Organ Preservation

In the previous two decades, clinically applied cold crystalloid cardioplegic solutions were used to preserve the porcine donor hearts. Byrne and McGregor assumed that an increased sensitivity of the porcine heart to ischemia/reperfusion injury was a major contributor to PCXD [36]. Although PCXD was overcome in the heterotopic thoracic xHTx model [43] and in singular cases of orthotopic xHTx experiments [21], consistent survival could not be achieved using this conservation technique. In 2016, Steen et al. developed a cold, non-ischemic preservation method with an oxygenated, hyperoncotic, erythrocyte containing cardioplegic solution, with which pig hearts were successfully preserved for up to 24 h [44]. The Munich group adopted this preservation method for orthotopic xHTx: after cardiopulmonary bypass had stopped, cardiac function was preserved, and inotropic support was reduced to a minimum [23, 44]. Several explanations have been postulated to be responsible for the results after non-ischemic porcine heart preservation: addition of oxygenated erythrocytes to minimize myocardial ischemia/reperfusion injury, hypothermia to reduce metabolic needs, continuous delivery of nutrients and removal of toxic metabolites, high oncotic pressure of the preservation medium and strict pressure-/flow-controlled coronary perfusion to inhibit edema formation and reduce capillary damage, and lastly, physiological levels of catecholamines, cortisol and thyroid hormones for maintenance of myocardial energy stores [45–48].

After several discouraging experiments with static ischemic preservation [24], Mohiuddin’s group also adopted the non-ischemic preservation technique [26]. Additionally, ischemic preservation with blood cardioplegia/Del Nido solution yielded promising results [27, 49], especially when the ischemic periods were kept very short [50].

## Immunosuppressive Therapies

The addition of CD40/CD40L costimulation blockade to an immunosuppression based on MMF, steroids and preoperative T and B cell depletion (ATG, rituximab) was an important step for prolonging xenograft survival after heterotopic abdominal and orthotopic pig-to-NHP xHTx [11, 23]. The first study showing that CD40/CD40L costimulation blockade was superior to conventional immunosuppression was done by Bühler et al [51] in 2000, and it was first used in xHTx by Kuwaki et al. in 2005 [5]. Since then, several experimental antibodies have been tested in preclinical xenotransplantation studies (reviewed in detail in [52]). Whereas initial results in heterotopic xHTx with anti-CD40L antibodies (clone 5C8H1) were very promising [53], first-generation antibodies proved to be thrombogenic in clinical trials due to activation of thrombocytes via binding to the FcγRII receptor [54] and were subsequently abandoned. Most recent results in preclinical orthotopic cardiac xenotransplantation were achieved with the chimeric anti-CD40 antibody clone 2C10R4 [25, 26]. For clinical use, a humanized version of this antibody has been developed by Kiniksa Pharmaceuticals (KPL-404, abiprubart), which completed a phase I trial [55] and recently commenced a phase II clinical trial for treatment of Sjögrens disease; this antibody was also applied in the first clinical xenotransplantation of a porcine heart into a human [28]. Several other pharmaceutical companies have antiCD40/CD40L antibodies in their pipelines (overview in [56, 57]). Until now, however, none of these antibodies have been approved for clinical use.

## Immunomodulatory Therapies

After pig-to-NHP xenotransplantation, systemic inflammation reactions were observed - termed “Systemic Inflammation in Xenograft Recipients” (SIXR) -, defined by an increase in inflammatory markers (C-reactive protein, histones, serum amyloid A, D-dimer, cytokines, chemokines) and a decrease in free triiodothyronine [58, 59]. While it is assumed that SIXR has a negative impact on xenograft survival by promoting coagulation activation and adaptive immune response [58], the exact role and mechanisms of SIXR are not fully understood. For orthotopic xHTx specifically, it has been postulated that the exposure to cardiopulmonary bypass has an additional detrimental effect [60]; this hypothesis is not generally accepted [61], however. To attenuate inflammation reactions after orthotopic xHTx, complement inhibitory drugs (cobra venom factor or C1 esterase inhibitor) and various immunomodulatory drugs (interleukin (IL) 1 and IL6 receptor blockers, tumor necrosis factor (TNF) α inhibitors) have been added to existing immunosuppressive regimens [23, 25–27, 62]. Furthermore, glycocalyx shedding, a surrogate parameter for endothelial dysfunction and inflammation, was only marginal under anti-inflammatory therapy [63]. It remains unclear, however, to what extend each of these anti-inflammatory substances contribute: for instance, IL6 receptor blockers have been shown to bind to baboon but not to pig IL6 receptors. Circulating IL6 - which is increased under treatment with IL6 receptor blockers [64] - could possibly moderate detrimental effects in the graft by activating porcine cells [65].

Another approach to prevent SIXR is the addition of anti-inflammatory transgenes to the porcine donor genome, such as human hemeoxygenase-1 (*hHMOX1*) or human tumor necrosis factor α-induced protein 3 (*hA20*), as was done in some of the recent studies [24, 26]. Further knowledge is however needed to clarify the potential benefit of these modifications.

Interestingly, cold non-ischemic preservation has also immunomodulatory effects [66]: after 8 h of *ex vivo* hypothermic cardioplegic perfusion, myocardial tissue was significantly immunodepleted, whereas the perfusate displayed a pro-inflammatory cytokine/chemokine pattern; the following heterotopic heart allotransplantation showed reduced leucocyte infiltrations in the transplant, and the graft’s viability was improved compared to controls. These results demonstrate a potential beneficial effect of cold non-ischemic preservation beyond sole reduction of ischemia/reperfusion, deserving further research in the xenotransplantation setting.

## Growth Inhibition

Extensive overgrowth of the donor heart was first described in a series of heterotopic thoracic pig-to-baboon xHTx experiments [43, 67], and subsequently, in orthotopic xHTx [23]. Graft overgrowth had also been observed after kidney transplantation between species of different body and organ sizes [68]. Besides genetic determination [69] (an intrinsic or donor-specific factor), several extrinsic (recipient-specific) factors have been described to influence cardiac growth after xHTX: nutrition [69], levels of growth hormone (GH), insulin-like growth factor 1 (IGF1) [70], hormones (thyroid hormones, vascular endothelial growth factor, insulin, catecholamines, endothelin, angiotensin), and mechanical stress/strain [71]. Interestingly, extensive cardiac overgrowth did not occur in the heterotopic abdominal pig-to-baboon xHTx model despite intrinsic mismatch of growth rate and organ size [11]: this is possibly due to the lack of relevant afterload in this non-working model (reviewed in [72]), leading to myocardial atrophy [73] and thereby counteracting the intrinsic growth of the graft [74]. By contrast, in the orthotopic model, the juvenile pig heart needs to adapt to an unphysiologically elevated afterload in an adult baboon [75]; elevated afterload is known to trigger myocardial hypertrophy [76, 77].

Untreated, this (mal-)adaptive myocardial hypertrophy leads to a phenomenon termed “xenogeneic Hypertrophic Obstructive Cardiomyopathy” (xHOCM) and eventually to graft dysfunction and graft loss [74]. Two different approaches have been described to prevent cardiac overgrowth: administration of growth inhibitory drugs or genetic modification of the donor animals. The Munich group administered a combination of a mTOR inhibitor (Temsirolimus), antihypertensive drugs (beta-blocker and ACE-Inhibitors) and fast steroid tapering to counteract intrinsic growth and attenuate cardiac remodeling [23]. Cleveland et al. used a similar approach by using an immunosuppressive regime based on rapamycin instead of MMF and fast steroid tapering; they also observed postoperative periods of severe hypertension, which were treated with milrinone and esmolol [27]. In these drug regimens, the mTOR inhibitor seems to be the most important substance: it has been shown to reduce and prevent cardiac hypertrophy in pressure overloaded animals [78, 79] as well as after human cardiac allotransplantation [80, 81]. By contrast, Mohiuddin et al. did not use drugs to inhibit graft growth, but 10fold genetically modified pigs, which lacked growth hormone receptors (*GHR-KO*) [26]; pigs with *GHR-KO* have been shown to grow slower and smaller than wild-type German Landrace pigs [82]. It has been proposed that a reduction of local myocardial IGF-1, produced by syngeneic resident macrophages, may play an additional role in inhibiting myocardial hypertrophy after xHTx with *GHR-KO* donor pigs [83, 84]. With growth inhibition, whether by drugs or genetic modifications, survival times of up to 6–9 months were achieved [23, 26, 27], thus emphasizing the tremendous importance of growth control after orthotopic xHTx. Especially for clinical use in adult humans, smaller donor races such as Auckland Island pigs are a future alternative to control growth [85], as undesirable effects associated with growth inhibitory drugs or genetic modifications can be avoided.

It must be noted that also rejection episodes [11, 26], as well as inflammation [74, 86] have been described to cause enlargement of the heart, mediated by myocardial edema, cellular infiltrate and/or hemorrhage. To what extend this contributes to chronic graft overgrowth after orthotopic xHTx is unknown; however, acute myocardial edema due to a rejection episode certainly has the potential to further damage an already hypertrophic xenograft.

## Avoiding PCMV/PCR

First described after pig-to-baboon kidney xenotransplantation [87, 88], infection of porcine donors with porcine cytomegalovirus – in fact a porcine roseolovirus (PCMV/PRV) – has been associated with significantly reduced graft survival after orthotopic pig-to-baboon xHTx [86, 89]. The underlying pathomechanism is still largely unknown, but it is assumed that PCMV/PRV infection causes an increase in levels of IL6, TNFα and tissue plasminogen activator inhibitor (tPA-PAI-1) complexes, suggesting a complete loss of the pro-fibrinolytic properties of endothelial cells, eventually leading to multiorgan failure of the recipient baboon [86, 90]. It has also been assumed that SIXR could be (at least in part) a reaction to PCMV/PRV [91, 92]. There is no effective antiviral treatment or vaccination, so xHTx of organs from PCMV/PRV infected donors must be strictly avoided (e.g., by motherless rearing [93] and rigorous testing protocols [90, 94]). In a recent retrospective analysis, the donors’ PCMV/PCR status did not significantly affect the outcome after orthotopic pig-to-baboon xHTx, with maximum recipient survival after transplantation of PCMV/PCR positive hearts of 225 days [95]. More data is needed, however, to confirm these preliminary data before the policy towards PCMV/PCR should be revisited.

## Conclusion

In the last 30 years, there has been significant progress in orthotopic pig-to-NHP xHTx, with recipient survival increasing from a few hours in 1994 to several months in 2024. Recent improvements in donor genetics, organ preservation, immunosuppressive and immunomodulatory treatments, donor organ growth inhibition and prevention of PCMV infection have led to consistent graft survival, thereby fulfilling the recommendations of the ISHLT as a prerequisite for a pivotal clinical trial. Some questions still remain, but a clinical application has never been closer than today.
